# Mitochondrial genome evolution in parasitic plants

**DOI:** 10.1186/s12862-019-1401-8

**Published:** 2019-04-08

**Authors:** Athanasios Zervas, Gitte Petersen, Ole Seberg

**Affiliations:** The Natural History Museum of Denmark, Faculty of Science, University of Copenhagen, Sølvgade 83, opg. S, DK-1307 Copenhagen K, Denmark

**Keywords:** Phylogeny, Parasitic plants, Substitution rates, Evolution, Parasitism, Balanophoraceae, Viscaceae, Mitogenome

## Abstract

**Background:**

Parasitic plants rely on their host to cover their nutritional requirements either for their entire life or a smaller part of it. Depending on the level of parasitism, a proportional reduction on the plastid genome has been found. However, knowledge on gene loss and evolution of the mitogenome of parasitic plants is only available for four hemiparasitic *Viscum* species (Viscaceae), which lack many of the mitochondrial genes, while the remaining genes exhibit very fast molecular evolution rates. In this study, we include another genus, *Phoradendron,* from the Viscaceae, as well as 10 other hemiparasitic or holoparasitic taxa from across the phylogeny of the angiosperms to investigate how fast molecular evolution works on their mitogenomes, and the extent of gene loss.

**Results:**

Our observations from *Viscum* were replicated in *Phoradendron liga*, whereas the remaining parasitic plants in the study have a complete set of the core mitochondrial genes and exhibit moderate or only slightly raised substitution rates compared to most autotrophic taxa, without any statistically significant difference between the different groups (autotrophs, hemiparasites and holoparasites). Additionally, further evidence is provided for the placement of Balanophoraceae within the order Santalales, while the exact placement of Cynomoriaceae still remains elusive.

**Conclusions:**

We examine the mitochondrial gene content of 11 hemiparasitic and holoparasitic plants and confirm previous observations in Viscaceae. We show that the remaining parasitic plants do not have significantly higher substitution rates than autotrophic plants in their mitochondrial genes. We provide further evidence for the placement of Balanophoraceae in the Santalales.

**Electronic supplementary material:**

The online version of this article (10.1186/s12862-019-1401-8) contains supplementary material, which is available to authorized users.

## Background

Parasitic plants comprise around 1% of all angiosperm species (http://parasiticplants.siu.edu/ParPlantNumbers.pdf). Depending on their photosynthetic ability, they are categorized as hemiparasites (facultative or obligatory) and holoparasites. Current phylogenetic evidence suggests that parasitism has evolved independently at least 12 times [1, 2], offering an excellent framework for studies of correlation between parasitism and genomic evolution. While a series of papers have recently focused on the correlation between levels of parasitism and loss of key photosynthesis genes in the plastome [3–5] and an instance of a possible complete loss of the plastome has also been reported in the holoparasite *Rafflesia lagascae* Blanco [6], comparatively little attention has been given to mitochondrial evolution.

The plastome of a typical, autotrophic angiosperm is circular, of a rather uniform size, and has a relatively conserved structure, with two unique regions separated by two inverted repeats, as well as a conserved gene content of ca. 40 protein coding genes, four rRNA genes and approximately 20 tRNA genes [7–9]. However, plastomes of the numerous parasitic angiosperms that have been sequenced show a pronounced variation both in size, structure and gene content. Not unexpectedly, there is a clear correlation between the type of parasitism and the level of plastome reduction and modification, e.g. plastomes of the holoparasitic members of Orobanchaceae [10], *Cuscuta* L. [3, 4], *Pilostyles* Guil. [11], *Rafflesia lagascae* [6]*, Hydnora visseri* Bolin, E.Maass & Musselman [12], and others, are much more reduced and modified than plastomes of hemiparasitic members of Orobanchaceae [5] and Viscaceae [13]. Similar evolutionary plastome reductions have been described in achlorophyllous mycoheterotrophic plants, which have either functionally or completely lost most or all genes involved in photosynthesis, e.g., *Neottia nidus-avis* (L.) Rich. [14], *Corallorhiza striata* Lindl. [15], *Petrosavia stellaris* Becc. [16], and several members of Ericaceae [17]. In hemiparasitic plants, capable of photosynthesis, major plastome reductions would not be expected, but nevertheless a recent study of the Santalales, and specifically on the hemiparasitic genera *Osyris* L. and *Viscum* showed that their plastomes are reduced by 10–22% compared to *Vitis* [13] the closest autotrophic relative sequenced.

Evolution of the mitochondrial genomes, henceforth mitogenomes, which are involved in basic cell metabolism, was not expected to be influenced by parasitism. Mitochondria are essential producers of the energy (ATP) of the cell and harbour key metabolic genes that control respiration, stress responses, and additionally defence against microorganisms and aspects of programmed cell apoptosis. Compared to plant mitochondrial genomes, animal mitochondrial genomes are in general fairly small in size (ca. 16 kb long), uniform in structure (circular) and gene content, encoding 22 tRNA genes, two rRNA genes and usually 13 protein coding genes of the respiratory chain complexes I: *nad1*, *nad2*, *nad3*, *nad4*, *nad4L*, *nad5*, *nad6*, III: *cob*, IV: *cox1*, *cox2*, *cox3*, and V: *atp6*, and *atp8* [18]. In contrast, plant mitogenomes exhibit profound differences: Their size is 10 to almost 1000 times larger than their animal counterparts, ranging from less than 200 kb to some 11.3 mb as in the case of *Silene conica* L. [19]. This has been partly attributed to the presence of larger introns, of more repeated sequences, and stretches of DNA transferred either from the nuclear and chloroplast genome, or from bacteria and viruses that have infected the plant [19, 20]. Electron microscopy and DNA sequencing has revealed that plant mitogenomes may be circular, linear, branching and comprising one or more molecules, as is the cases in e.g. *Silene conica* [19], *Oryza sativa* L. [21] and *Brassica napus* L. [22]. Regarding their standard gene content, plant mitogenomes contain all the genes present in the animal mitogenomes, with additional subunits in respiratory complex I: *nad7, nad9*, V: *atp1, atp4, atp9*, as well as in complex II: *sdh3, sdh4*. In addition to the genes of the respiratory chain, plant mitogenomes contain a maturase-related protein gene (*matR*), genes encoding for proteins of the small and large subunit of the ribosomes (*rps* and *rpl* genes) and the genes *ccmB, ccmC, ccmFc* and *ccmFn* that are involved in the cytochrome C biogenesis pathway. All genes from complexes I, III, IV, and V, which are considered as the core mitochondrial genes, are present in all hitherto sequenced autotrophic land plants [23], while the remaining genes comprise a variable part of the plant mitogenome. This was also observed in the holoparasitic plants *Rafflesia lagascae* [6] and *Cynomorium coccineum* [24], and similar results have been reported for three hemiparasitic and three holoparasitic Orobanchaceae, all of which possessed a complete core mitochondrial gene set [25]. However, recent studies on four species of *Viscum* showed a surprising reduction in gene content compared to all other known angiosperm mitogenomes [26, 27]. This observation prompts the obvious question, whether a loss of genes in the mitogenome similar to that observed in the Viscaceae is found in other parasitic lineages as well.

In addition to extensive gene loss, a very high level of sequence divergence of the remaining key genes was also observed in the four species of *Viscum* [26, 27]. Especially the genes in respiratory complex V, *atp1, apt6, atp8,* and *atp9*, have very high substitution rates compared to other plants. However, a few genes, e.g. *matR* in *V. album* L., retain a low substitution rate. In general, mitochondrial genomes have low substitution rates, lower than what is found in the chloroplast genomes, and 10–20 times lower compared to the plant nuclear genomes [28]. Often, higher substitution rates in the mitogenome are accompanied by higher substitution rates in the other two plant genomes, and are inferred to be a characteristic of the species that exhibits them, e.g. in certain species of *Plantago* L. [29]. However, it has been shown later that certain plant genera, like *Pelargonium* L. H. Bailey, have high substitution rates only in their mitogenomes [30]. Comparison of nuclear small-subunit rDNA from partially autotrophic, chlorophyllous hemiparasites, and achlorophyllous holoparasites showed that the latter have much higher substitution rates than the former [31]. Higher chloroplast substitution rates have been suggested to be correlated with species richness, as in the case of the Proteaceae [32]. Early theoretical models have suggested that parasites should evolve much faster than their hosts as a response to the host-parasite arms race [33]. A comparison of parasitic plants to their closest non-parasitic relatives showed that the first tend to have higher substitution rates across all three genomes [34]. Thus, in this study we also want to test how general the significantly higher substitution rates seen in the mitochondrial genes of *Viscum* are in parasitic plants and whether higher substitution rates are correlated to levels of parasitism or is clade specific.

## Materials and methods

### Plant material

Plant material was obtained from Herbarium C and the silica gel collection of the Natural History Museum of Denmark, University of Copenhagen, Denmark. To the best of our knowledge all plants have been legally collected. Details on the plants examined in the study (including authors) are listed in Table 1. The vouchers are all deposited in Herbarium C. Author names are according to the International Plant Names Index (www.ipni.org).

**Table 1:** Parasitic plants examined in this study

#	Project ID	Family	Species	Collector	Date of collection	Area of collection	Type of parasitism
1	C3122	Boraginaceae	*Pholisma sonorae* (Torr. ex A. Gray) Yatsk	R. Thome 52,167	1978	USA, California, Algodones dunes	Holoparasitic
2	C3123	Lauraceae	*Cassytha pubescens* Schltdl.	Whibleg 9833	1985	Australia, South Australia	Hemiparasitic
3	C3124	Krameriaceae	*Krameria lanceolata* Torr.	Museelman 4841	1975	USA, Florida	Hemiparasitic
4	C314	Balanophoraceae	*Langsdorffia hypogaea* Mart.	P. Barbour 2646	1978	Peru, Province Bagua, 12 km E of da Peca	Holoparasitic
5	C1935	Orobanchaceae	*Lathraea squamaria* L.	G. Petersen & O. Seberg	25/04/05	Denmark, Virum, Geels Skov	Hemiparasitic
6	C3089	Orobanchaceae	*Lathraea cladestina* L.	H. Æ. Pedersen 811	16/05/14	Spain, Alava, Done Bikendi Havana	Hemiparasitic
7	C3127	Loranthaceae	*Loranthus europaeus* Jacq.	Carlos Reif	22/02/15	Czeck Republic, Kutna Hora	Hemiparasitic
8	C3151	Cynomoriaceae	*Cynomorium coccineum* L.	F. N. Rasmussen	01/05/15	Portugal	Holoparasitic
9	C3152	Cytinaceae	*Cytinus hypocistis* L. ssp*. clusii* Nyman	F. N. Rasmussen	05/05/15	Portugal, Algarve, 13.2 km NE of Albufeira	Holoparasitic
10	C3158	Cytinaceae	*Cytinus hypocistis* L. ssp*. hypocistis*	G. Petersen	30/05/15	Italy, Liguria	Holoparasitic
11	C2482	Viscaceae	*Phoradendron liga* Eichler	O. Seberg & G. Petersen	02/11/08	Argentina, Salta	Hemiparasitic

### DNA extraction

DNA was extracted using two different protocols depending on the type of the sample; one for the herbarium samples and one for the silica gel samples. DNA was extracted from the herbarium samples in the clean lab of the Natural History Museum of Denmark, University of Copenhagen, Denmark, using the ancient plant DNA extraction protocol by Wales et al. [35]. DNA was extracted from the silica gel samples using the DNA Plant Minikit (Qiagen) according to the manufacturer’s instructions, with the addition of a Proteinase K treatment step, for 2 h at 65 °C, immediately after the bead-beating step (Qiashredder). Details on tissue type, protocol, cleaning procedures and mass used for DNA extraction are given in the Additional file 1: Table S1.

### Library preparation

The DNA extracted from the herbarium samples was already broken down into very small fragments, whereas the DNA from the silica gels samples was sheared using a Bioruptor (Diagenode). 100 μl dilutions of 10 ng/μl DNA concentration were sheared using the following conditions: 5 cycles of 15 s ON, 90 s OFF. The sheared DNA was then run on a 2% agarose gel with a 100 bp DNA ladder (Thermo Scientific), to check the size variation of the DNA fragments. For all samples we obtained DNA fragments in the range of 200–600 bp.

For the resulting 11 samples, the NEBNext DNA Sample Prep Master Mix Set 2 (New England Biolabs, Ipswich, Massachusetts) using blunt-end adapters specified by Meyer and Kircher [36] was used to prepare the fragments for inserting the adapter sequences and unique barcodes. In order to determine the number of cycles needed for each library, qPCRs were performed on 1:40 dilutions of each DNA sample, using the SYBR Green Master Mix 1 (Agilent Technologies) on an Mx3500p qPCR machine (Agilent Technologies). For each sample a common forward primer and a unique, custom, reverse index primer was used in PCRs. Libraries were amplified 10–14 cycles in a total volume of 100 μL, containing 5 U AmpliTaq Gold polymerase (Applied Biosystems, Foster City, CA), 1× AmpliTaq Gold buffer, 2.5 mM MgCl_2_, 0.4 mg/mL bovine serum albumin (BSA), 0.2 mM of each dNTP, 0.2 μM IS4 forward primer, 0.2 μM indexed reverse primer, and 20 μL DNA library template. Following amplification, libraries were purified using a QIAquick PCR Purification kit (Qiagen, Hilden, Germany), according to manufacturer’s instructions. DNA was eluted in 32 μL EB buffer and the column was incubated for 10 min at 37 °C prior to centrifugation. The libraries were first quantified on a Qubit 2.0 (Life Technologies, Carlsbad, CA) using a dsDNA high sensitivity assay, and then run on a TapeStation 2200 using the high sensitivity tapes (Agilent, Santa Clara, CA) to determine the average insert size and molarity of each library. Then two sample pools were created, one for the samples from the herbarium and another for the samples from silica gel.

### Next generation sequencing

The sequences were obtained using Illumina sequencing on a Hiseq 2500 at the National High-Throughput DNA Sequencing Centre of the University of Copenhagen, Denmark. Details on the run, chemistry, number of raw reads obtained and number of reads that passed quality control are given in Table 2. Sequences for *Phoradendron liga* were obtained from a previous investigation, where 150 bp pair end sequencing was used. For the silica gel samples 100 bp pair end sequencing was chosen, while for the herbarium samples 100 bp single end sequencing is the preferred method as the DNA is already degraded rendering pair end sequencing sometimes inefficient.

**Table 2:** Sequencing information

#	Project ID	Species	Sequencing platform	Sequencing chemistry	Raw reads	QC Trimmed reads	Mitochondrial gene coverage
1	C3122	*Pholisma sonorae*	Illumina HiSeq 2500	100 bp Single End	14,993,887	14,838,255	7X
2	C3123	*Cassytha pubescens*	Illumina HiSeq 2500	100 bp Single End	129,061,347	127,650,167	81X
3	C3124	*Krameria lanceolata*	Illumina HiSeq 2500	100 bp Single End	14,392,427	14,102,890	7X
4	C314	*Langsdorffia hypogaea*	Illumina HiSeq 2500	100 bp Single End	8,584,595	8,118,411	31X
5	C1935	*Lathraea squamaria*	Illumina HiSeq 2500	100 bp Pair End	78,319,916	44,057,801	495X
6	C3089	*Lathraea clandestina*	Illumina HiSeq 2500	100 bp Pair End	54,446,884	31,074,675	92X
7	C3127	*Loranthus europaeus*	Illumina HiSeq 2500	100 bp Pair End	100,902,100	57,189,199	386X
8	C3151	*Cynomorium coccineum*	Illumina HiSeq 2500	100 bp Pair End	138,075,206	49,400,497	30X
9	C3152	*Cytinus hypocistis* ssp*. clusii*	Illumina HiSeq 2500	100 bp Pair End	138,075,206	66,503,764	901X
10	C3158	*Cytinus hypocistis* ssp*. hypocistis*	Illumina HiSeq 2500	100 bp Pair End	78,272,446	29,053,324	330X
11	C2482	*Phoradendron liga*	Illumina HiSeq 2500	150 bp Pair End	29,638,962	23,696,088	40X

### NGS data analysis

The obtained raw reads were trimmed for quality, adapters and unidentified nucleotides (Ns) using Adapter Removal [37]. The reads that passed the quality control were then imported into Geneious version R9.0.5 (Biomatters Ltd.). The reads from each taxon were mapped to the mitochondrial gene sets of 5 autotrophic taxa spread across the angiosperm phylogeny ( *Brassica carinata* L.A. Braun NC_016120 [38], *Vitis vinifera *L. NC_0122119, *Liriodendron tulipifera* L. NC_021152, *Salvia miltiorrhiza* NC_023209 and *Glycine max* NC_020455). The Geneious mapper was used with the following settings: Iterate 5 times, medium/low sensitivity, fast. Consensus sequences were then created using a strict criterion of 75% read agreement, calling gaps when the coverage was below 10x, using the Geneious consensus sequence tool. Additionaly, to recover divergent mitochondrial genes that would not be identified by mapping-to-reference runs, we employed de novo assemblies using SPAdes [39] with default settings. The resulting contigs were annotated as previously to identify those of mitochondrial origin and mapping-to-reference runs were employed as described above to check for mean coverage to use as proxy to identify more mitochondrial contigs which lacked annotations. Using relaxed search criteria on a local BLAST database comprised of the mitochondrial genes of *Brassica carinata, Viscum album* and *Viscum scurruloideum*, the presence/absence of mitochondrial genes was tested on the resulting contigs.

### Phylogenetic analysis and statistics

**Fig. 1:**
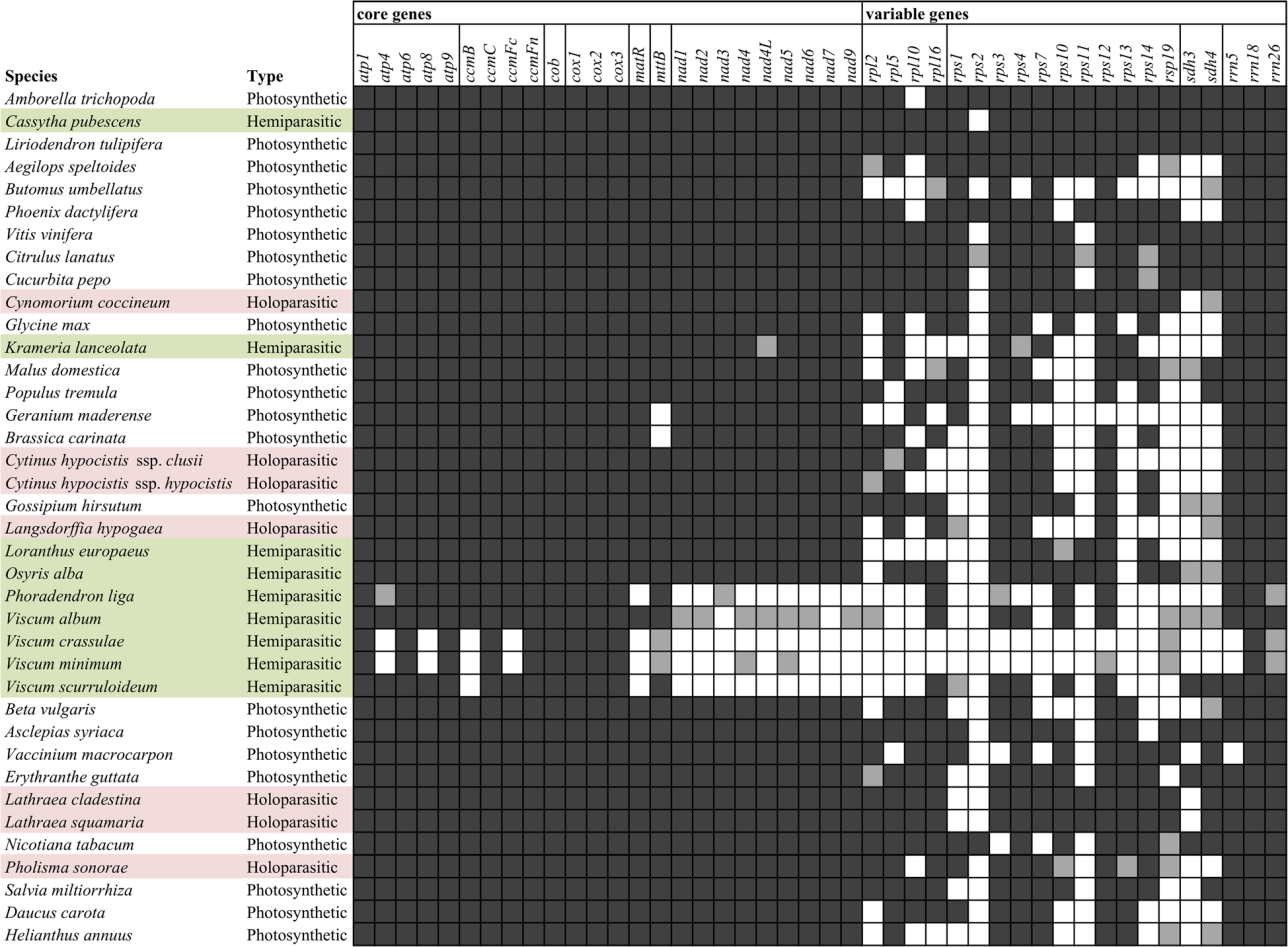
Protein-gene content of 38 angiosperms, including the 11 parasitic plants analysed in this study. Dark grey boxes indicate presence of a gene, light grey boxes indicate pseudogenes or partial genes, and white boxes indicate absence of the gene. Taxa in green show hemiparasitic and taxa in light red show holoparasitic plants

For the phylogenetic analysis, a custom database comprising all mitochondrial gene sequences of the 11 taxa analyzed in this study and of 27 non-parasitic taxa (Fig. 1; Additional file 2: Table S4), which represent all major orders in the angiosperm phylogeny, was created in Geneious. Our emphasis was to have at least one non-parasitic plant as closely related as possible to each of the parasitic plants. Each gene set was aligned using MAFFT v7.222 [40] under the following options: Algorithm FFT-NS-i × 1000, scoring matrix 200PAM / k = 2 and default gap open penalty and offset value, as implemented in Geneious R9.0.5. The overhangs of each alignment were then trimmed using the implemented function in Geneious (Tools- > Mask alignment- > More than 70% gaps), so that all sequences from each gene have the same length. The alignments of the core genes were then concatenated into the following groups of genes: 1) all_atp_genes, which includes *atp1*, *atp4*, *atp6*, *apt8*, and *atp9*, 2) all_ccm_genes, which includes *ccmB*, *ccmC*, *ccmFc*, and *ccmFn*, and 3) all_nad_genes, which includes *nad1*, *nad2*, *nad3*, *nad4*, *nad4L*, *nad5*, *nad6*, *nad7*, and *nad9.* Regarding the variably occurring ribosomal protein genes (*rpl* and *rps*) alignments were made for *rpl2*, *rpl5*, and *rpl16*, and *rps3* as the remaining genes were frequently missing from some of the 38 taxa. Finally, a concatenation of the mitochondrial core gene alignments, including *rps3, rpl2, rpl5,* and *rpl16* gave the MT_genes alignment with a length of 40,091 positions (29,036 positions without gaps).

In order to expand the taxonomic sampling size of our analysis we merged part of our data matrix with the one used by Qiu et al. [41], which contains mitochondrial gene sequences from 380 taxa, but only four mitochondrial genes, namely *atp1, rps3, matR* and *nad5*. Thus, we only used these four sequences from our smaller data set as well. The concatenated matrix from the Qiu et al. data set were separated into the four genes mentioned above, re-aligned with the sequences of our data set using MAFFT v.7222 with the same options as previously, the overhangs were trimmed and the sequences were concatenated. The final data set included 418 terminals and had a total length of 9076 positions.

Phylogenetic trees were constructed for 1) each mitochondrial gene (analyses 1 and 2) each concatenated group of aligned genes (analyses 2 and 3) for the concatenated alignment that includes all mitochondrial genes (MT_genes alignment) (analyses 3 and 4) for the concatenated sequences of the larger data set of the 418 terminals (analysis 4). All trees were visually inspected for significant topological conflicts. Phylogenetic analyses were performed in RAxML [42] using the GTR-Gamma substitution model, utilizing the Rapid Bootstrapping and search for best ML-scoring tree algorithm with 1000 bootstrap replicates, and using either *Amborella* Baill. (analyses 1–3) or *Cycas* L. plus *Zamia* L. (analysis 4) as the outgroup. The resulting tree files with bootstrap values were imported into FigTree (http:// tree.bio.ed.ac.uk/software/figtree/) for visualization.

Statistical analyses of the total number of substitutions on the phylogenetic trees were based on all mitochondrial gene sequences (analysis 3) and the large data set (analysis 4) was done in R (http://www.R-project.org), using the MASS [43], APE [44], Faraway [45], and Agricolae [46] libraries (Additional file 3: Code C1). The tree files in Newick format were used as input in both cases. One-way analysis of variance (1-way ANOVA) was performed grouping the plants into 1) four groups: autotrophic, hemiparasitic, holoparasitic plants and Viscaceae, and 2) three groups: autotrophic, hemiparasitic (including the Viscaceae), and holoparasitic plants. The significance of the analysis was verified by running Tukey HSD (Honestly Significant Difference) tests and the significant threshold value was *p* < 0.1.

## Results

### Mitochondrial gene content in parasitic plants

The vast majority of all hemiparasitic and holoparasitic plants analyzed in our study possess an almost complete mitochondrial core gene complement, where all genes from complex I (*nad1*, *nad2*, *nad3*, *nad4*, *nad4L*, *nad5*, *nad6*, *nad7* and *nad9*), complex III (*cob*), complex IV (*cox1*, *cox2* and *cox3*) and complex V (*atp1, atp4, atp6, atp8* and *atp9*) are present, along with the maturase-related protein gene (*matR*) and the genes *ccmB, ccmC, ccmFc* and *ccmFn* that are involved in the cytochrome C biogenesis pathway (Fig. 1).

*Krameria lanceolata* appears to be the only species to possess only a partial, not functional *nad4L* gene (197 nucleotides, 65% of the complete *Brassica carinata nad4L* gene, missing part of the 5′ sequence). With regards to complex II (*sdh3, sdh4*), there is not a distinct pattern of presence/absence. Hence, *Cassytha pubescens* harbours both genes; *Cynomorium coccineum* and *Langsdorffia hypogaea* only possess a partial *sdh4* gene, *Lathraea clandestina* and *L. squamaria* have a complete *sdh4* gene; while *Loranthus europaeus*, *Krameria lanceolata, Cytinus hypocistis* ssp. *clusii, C. hypocistis* ssp. *hypocistis* and *Pholisma sonorae* have lost both. The three rRNA genes appear to be present in all taxa in the analysis, except for *Phoradendron*, while the variable genes encoding for proteins of the small and large ribosomal subunits are absent in a random pattern. Hence, several parasitic taxa possess 5–6 out of the maximally 15 *rpl* and *rps* genes in addition to 1–3 pseudogenes, while others have 14 ribosomal protein genes, namely *Cassytha pubescens* and *Cynomorium coccineum*, both only lacking the *rps2* gene. This gene appears to be missing from all parasitic taxa of this data set, and also from many autotrophic plants. For the remaining genes encoding for proteins of the two ribosomal subunits in the 12 parasites sequenced here, *rpl2* is present in six taxa, *rpl5* in nine, *rpl10* in seven, while *rpl16* in just one; *rps2* is absent from all, while the rest of the *rps* genes vary from 5 to 6 to 10 per parasitic plant, without a clear pattern of loss.

A remarkable exception is the mitogenome of *Phoradendron liga*, which like species of *Viscum* lacks all *nad* genes, but also *ccmB*, *matR*, and possibly *atp4* and *rrn26*. Many ribosomal protein genes are also lost. The core mitochondrial genes show great divergence compared to “normal” plant mitochondrial genes. Hence, these genes show highest similarity to the respective ones from *Viscum album* (NC_029039), with *atp1* showing 81% identity to *V. album* and less than 75% to other taxa and *atp9* showing 92% identity to *V. album* when running BLASTN search. The remaining genes exhibit similarities ranging from 80 to 86%, to a variety of distantly related taxa, while the general identity between the respective genes of different taxa is higher than 92% (data not shown).

### Phylogenetic analysis

**Fig. 2:**
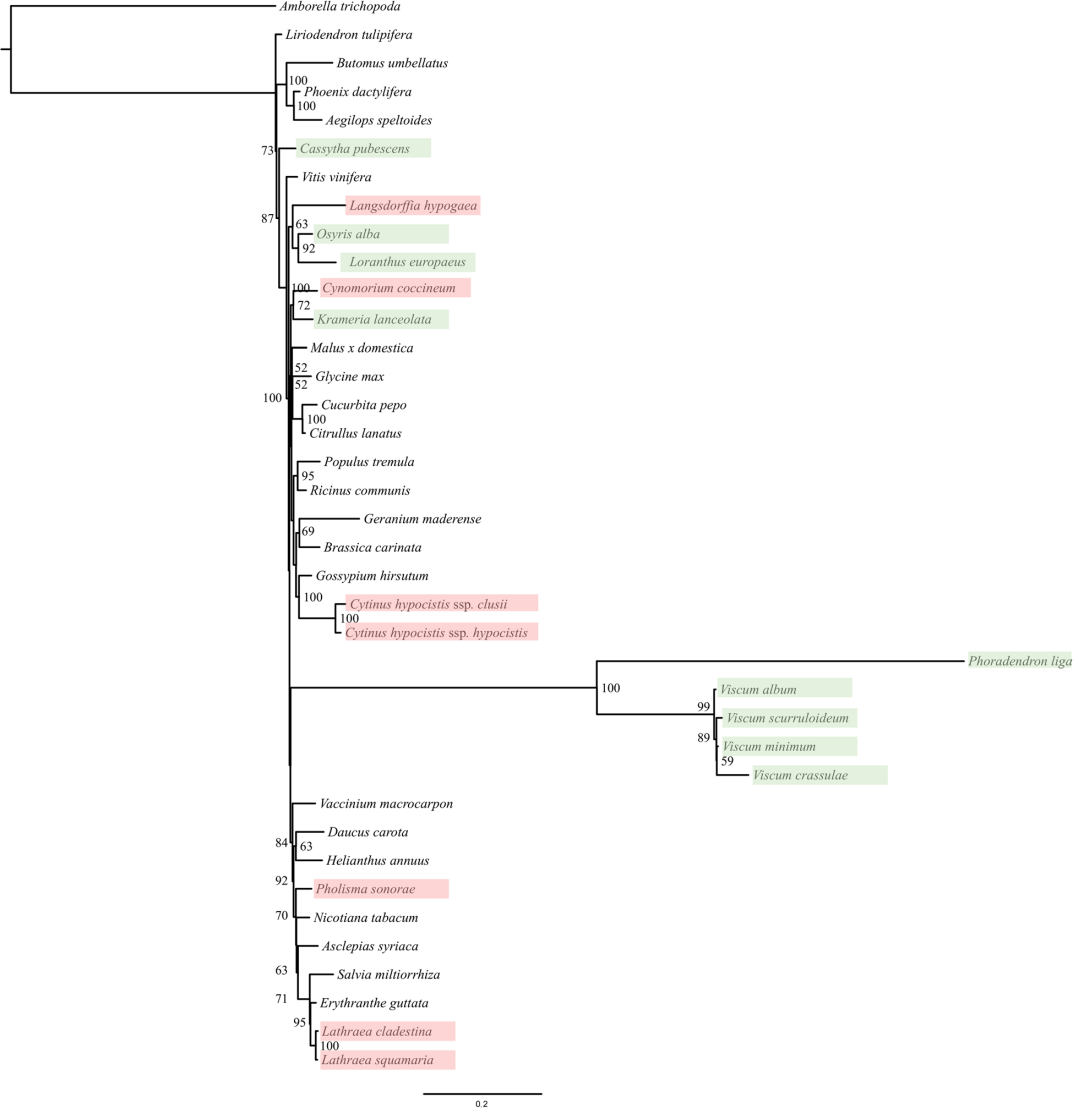
Phylogenetic tree of the 38 angiosperms included in the study. Branch length shows substitution rates, while bootstraps support values are shown on the base of each branch. Taxa in green show hemiparasitic and taxa in red show holoparasitic plants

The data set used in analysis 3 consisting of the total aligned and concatenated mitochondrial genes of just 38 taxa results in a tree with most species in their expected phylogenetic position (Fig. 2). Thus, *Lathraea clandestina* and *L. squamaria* cluster next to *Erythranthe guttata* (DC.) G. L. Nesom and *Salvia miltiorrhiza* Bunge in the order Lamiales, and *Pholisma sonorae* (Boraginales) is also placed within the Lamiids. *Loranthus europaeus* and *Langsdorffia hypogaea* cluster with *Osyris alba* forming the Santalales and *Phoradendron liga* clusters with high support (BS = 100%) in the Viscaceae, however the clade is not placed within the Santalales. The two *Cytinus hypocistis* subspecies cluster with *Gossypium hirsutum* L. in the order Malvales. *Krameria lanceolata* (Zygophyllales) is placed as the sister to *Cynomorium coccineum* (Saxifragales) but the clade has low support (BS = 72%). *Cassytha pubescens* (Laurales) branches off relatively near the root of the tree, but is not placed as the sister to the other representative of Laurales, *Liriodendron tulipifera*.

Expanding the analysis to a total of 418 terminals, while reducing the number of mitochondrial genes to four (analysis 4), (Additional file 4: Figure S1), most of the apparent misplacements seen in the smaller data set are corrected due to the denser taxon sampling, hence *Cassytha pubescens* is placed within the Laurales as sister to *Laurus* L. (BS = 67%), *Krameria lanceolata* clusters with *Guaiacum* L. (Zygophyllales) (BS = 100%), close to other representatives of the Fabiids, while *Pholisma sonorae* is placed within Boraginales as closely related to *Ehretia* P. Browne*, Hydrophyllum* L. and *Borago* L. (BS = 100%). Similarly, *Lathraea cladestina* and *L. squamaria* are well supported within the Lamiales as sister to *Erythranthe guttata* (BS = 99%). Both *Langsdorffia hypogaeae* (Balanophoraceae) and *Osyris alba* (Santalaceae) are placed within the Santalales, the latter as the well-supported sister to *Santalum* L. (BS = 96%). Additionally, *Loranthus europaeus* (Loranthaceae) is placed in the Santalales as a strongly supported sister to *Gaiadendron* G. Don (Loranthaceae) (BS = 98%). *Phoradendron liga* and the remaining Viscaceae form a strongly supported clade (BS = 100%), however still not included in Santalales, but placed within a well-supported clade including the Ericales (BS = 94%). *Cynomorium coccineum* (Cynomoriaceae) is places as the sister to a clade corresponding to the superasterids, but the relationship is unsupported.

### Substitution rates

Table 3 shows the substitution rates of each taxon for 1) all the mitochondrial genes, and 2) *atp1*, 3) *matR* and 4) *rps3*, respectively. It is evident that in this analysis the mean mitochondrial gene substitution rate is highly influenced by the fast evolving *atp1* gene. *Butomus umbellatus* L. has some of the fastest evolving *atp1, matR* and *rps3* genes, but the presence of the remaining slowly evolving mitochondrial genes lowers its mean substitution rate. The majority of the holoparasitic plants in this analysis tend to have higher substitution rates compared to the autotrophic ones, but *Geranium maderense* is evolving even faster still. It is notable that *Viscum album*, albeit having one of the fastest evolving and most divergent *atp* gene sets, has a slowly evolving *matR* gene. Among the representatives of Viscaceae analysed here, only *V. album* has a complete *matR* gene.

**Table 3:** Substitution rates of the *atp1, matR, rps3,* and all the mitochondrial genes of the 38 taxa of analysis 1 and 3. The taxa are ordered in ascending order based on the total substitution rates of all the mitochondrial genes

Species	*all_mt*	*atp1*	*matR*	*rps3*
*Amborella trichopoda*	0.45	0.45	0.45	0.45
*Liriodendron tulipifera*	0.460	0.463	0.464	0.465
*Cassytha pubescens*	0.484	0.511	0.468	0.478
*Vitis vinifera*	0.487	0.517	0.472	0.477
*Phoenix dactylifera*	0.491	0.477	0.479	0.486
*Citrullus lanatus*	0.500	0.516	0.496	0.493
*Ricinus communis*	0.502	0.516	0.490	0.488
*Malus x domestica*	0.503	0.534	0.490	0.499
*Nicotiana tabacum*	0.508	0.535	0.497	0.490
*Glycine max*	0.511	0.556	0.492	0.499
*Gossypium hirsutum*	0.511	0.531	0.486	0.510
*Pholisma sonorae*	0.511	0.538	0.495	0.520
*Osyris alba*	0.513	0.529	0.477	0.483
*Krammeria lanceolata*	0.513	0.542	0.496	0.500
*Vaccinium macrocarpon*	0.517	0.573	0.500	–
*Erythranthe guttata*	0.519	0.596	0.495	–
*Cucurbita pepo*	0.520	0.538	0.525	0.514
*Cynomorium coccineum*	0.521	0.547	0.500	0.531
*Lathraea squamaria*	0.521	0.599	0.491	0.507
*Lathraea clandestina*	0.522	0.594	0.491	0.508
*Asclepias syriaca*	0.522	0.569	0.502	0.502
*Brassica carinata*	0.525	0.560	0.507	0.537
*Populus tremula*	0.525	0.648	0.503	0.537
*Aegilops speltoides*	0.529	0.519	0.512	0.527
*Helianthus annuus*	0.529	0.533	0.492	–
*Daucus carota*	0.532	0.541	0.489	0.528
*Butomus umbellatus*	0.547	0.713	0.550	0.597
*Salvia miltiorrhiza*	0.548	0.598	0.493	0.511
*Cytinus hypocistis* ssp. *clusii*	0.553	0.554	0.520	0.648
*Cytinus hypocistis* ssp. *hypocystis*	0.561	0.583	0.534	0.577
*Langsdorffia hypogaea*	0.569	0.579	0.600	0.568
*Loranthus europaeus*	0.569	0.583	0.534	0.580
*Geranium maderense*	0.593	0.614	0.524	0.880
*Viscum album*	1.199	1.377	0.492	1.345
*Viscum minimum*	1.203	1.382	–	–
*Viscum scurruloideum*	1.210	1.381	–	1.347
*Viscum crassulae*	1.255	1.383	–	–
*Phoradendron liga*	1.621	1.681	–	–
Mean	0.622	0.669	0.500	0.581
Median	0.521	0.554	0.495	0.511

**Fig. 3:**
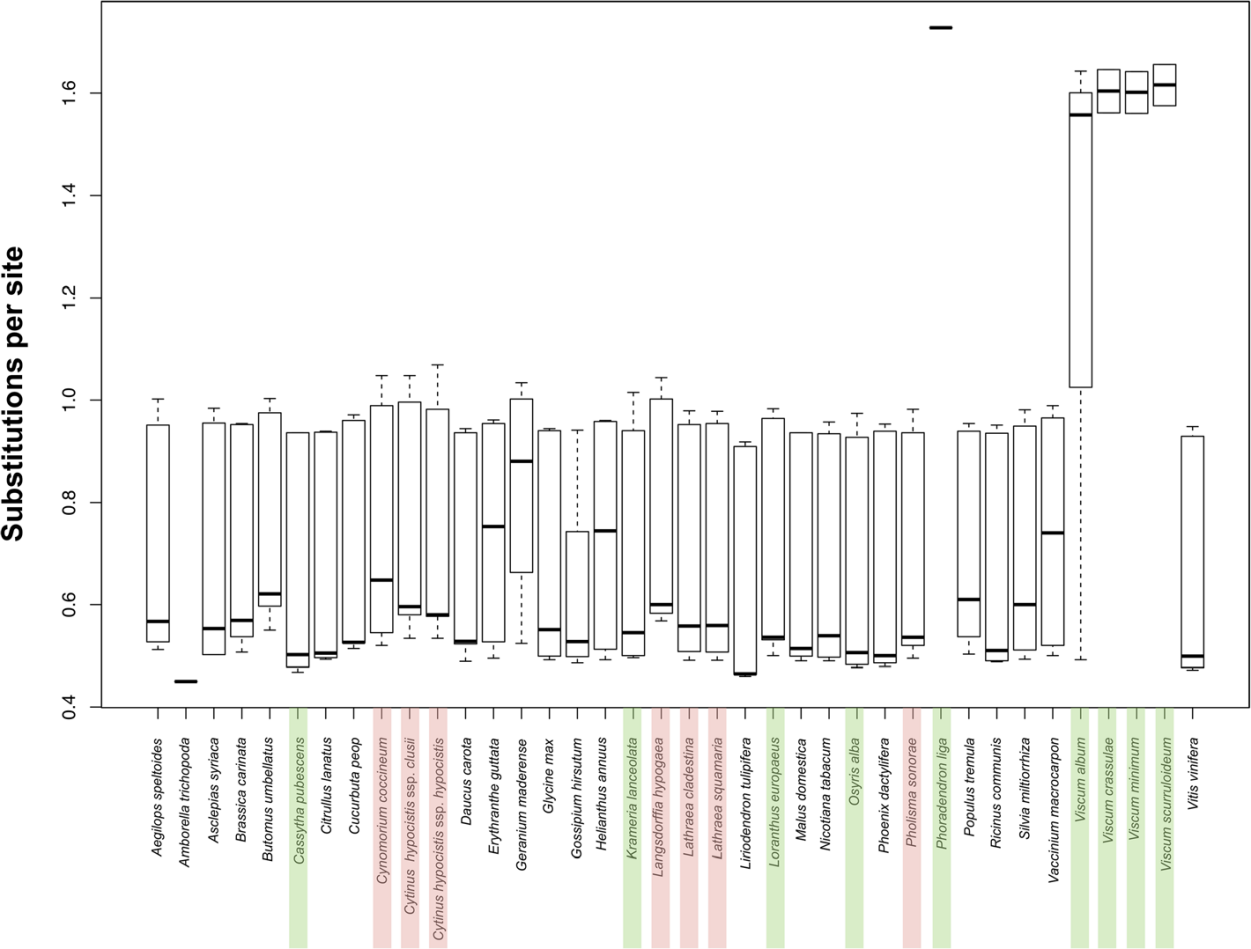
Barplot with the substitution rates of the mitochondrial genes of the 38 taxa in analysis 1. Taxa in green show hemiparasitic and taxa in red show holoparasitic plants

Figure 3 shows the substitution rates of all mitochondrial genes of the 38 taxa used in Table 3. Black lines depict the mean substitution rates of each mitogenome, with regards to its coding sequences, only. As the outgroup, *Amborella* has a fixed value. This peculiarity is due to a bug in RaXML as has been addressed by the developer of the program, yet since all distances are calculated from the root of the tree, and since *Amborella* is not the focus of this study, this bug is insignificant to the downstream analysis of the results. *Phoradendron liga* also has a fixed value due to the lack of the *matR* and *rps3* genes used in the analysis, while its *atp1* and all_mt_genes rates are quite similar. It is evident that there are no great differences between the taxa, irrespective of whether they are autotrophic, hemiparasitic or holoparasitic. A profound exception is, as shown earlier [26, 27], the Viscaceae, with *Phoradendron liga* showing the same pattern as the *Viscum* spp. The few sequences obtained from this hemiparasite show very low similarity to the homologous sequences of other plant species, thus for *atp1* the degree of similarity varies from 49 to 73%, for *atp9* from 37% to 82%, for the *cox* genes from 62 to 71% and for *rps12* from 27 to 41%. The boxplot also demonstrates that there are a few autotrophic plants, which show relatively higher substitution rates than the parasitic plants.

Expanding the analysis (analysis 4) reveals that across the angiosperms, most taxa show mean substitution rates in the vicinity of 0.65 to 0.75 substitutions per site (Supplementary Material, Figure S1). However, *Acorus* L., *Geranium* L., *Hydrolea* L. and others also exhibit high substitution rates, similar to those found in Viscaceae.

How significant is the difference seen in the Viscaceae, compared to the other taxa? Additional file 5: Table S3 shows the results of the 1-way ANOVA in the small data set, where the samples are put into four categories: autotrophic, hemiparasitic, holoparasitic plants and the Viscaceae. First, it shows once again that *atp1* (Additional file 5: Table S3a) evolves faster compared to *matR*, *rps3*, and *nad5*, with mean substitution rates for the 3 groups (autotrophic, hemiparasitic, holoparasitic) ranging from 0.53 to 0.58 (excluding the Viscaceae), followed by *matR* (Additional file 5: Table S3b), with mean substitution rates ranging between 0.49 and 0.52, and *rps3* (Additional file 5: Table S3c), with mean substitution rates ranging between 0.47 and 0.56. The mean substitutions per site for all the mitochondrial genes shown in the four groups are 0.51, 0.51, 0.55 and 1.3 for the autotrophic, hemiparasitic, holoparasitic plants and Viscaceae, respectively (Additional file 5: Table S3d). The Tukey HSD test reveals no statistically significant difference between the autotrophic, hemiparasitic and holoparasitic plants. On the other hand, Viscaceae show statistically significant difference compared to the other three groups (*p* < 0.01). Similarly, when Viscaceae are included in the hemiparasitic group (data not shown), they still show significant differences compared to the remaining two groups (*p* < 0.01), which can be attributed to the very small sample size and homogeneity of the group (nine taxa).

**Fig. 4:**
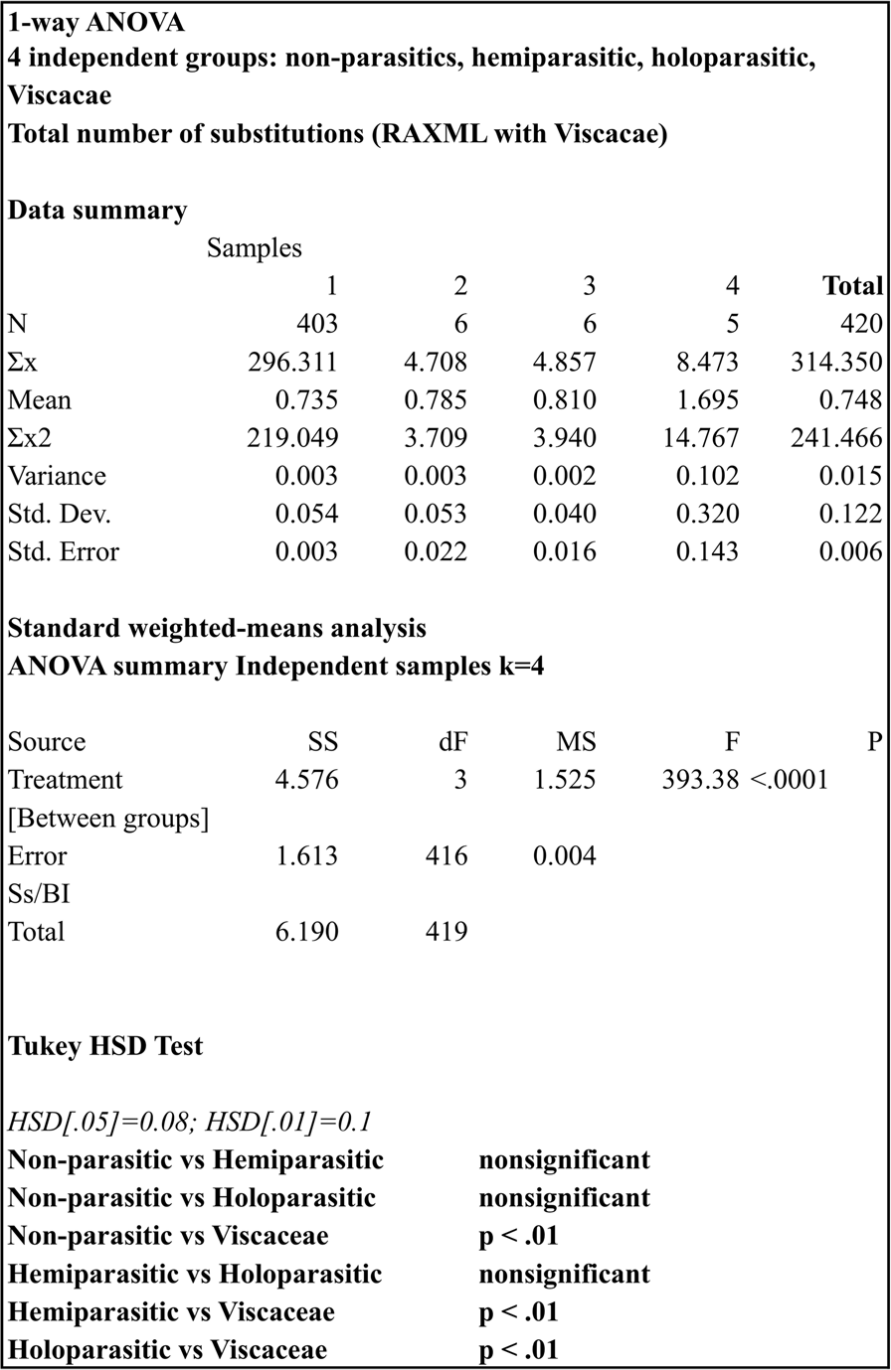
Analysis of variance of the substitution rates in the large data set of the mitochondrial genes (analysis 4)

Performing the same analyses using the expanded Qui et al. data set, we once again see that Viscaceae have significantly higher substitution rates than the autotrophic, hemiparasitic and holoparasitic plants (*p* < 0.1) (Fig. 4). However, comparing the individual taxa to each other, *Viscum album* is not statistically different from non-parasitic taxa such as *Acorus, Geranium, Podocarpus* Pers. and others (Additional file 6: Table S2). *Viscum album* is currently the only representative of Viscaceae that still possesses a *matR* gene, and the low substitution rate of this gene lower the total substitution rate of the mitogenome of the species.

## Discussion

### Mitochondrial gene loss in most parasitic plants is confined to the variable part of the mitogenome

Nearly all angiosperm mitogenomes assembled thus far have a full core gene complement, comprising of 24 genes. Exceptions are genera like *Vigna* Savi, which is missing the *cox3* gene [20], and *Silene* L., which is missing the *ccmFc* gene [19]. The latter, despite having one of the largest plant mitogenomes known (11.3 mb), has also lost all but one or two of the variable genes (four *rpl*, 11 *rps* and two *sdh* genes). Recently it was found that *Viscum* possesses one of the smallest and most dramatically reduced plant mitogenomes investigated so far [26, 27] .

In the present investigation, we sequenced five hemiparasitic and seven holoparasitic plants and extracted all known plant mitochondrial genes. Most of them possessed a full mitochondrial core gene complement. One exception is the hemiparasite *Krameria lanceolata*, which possesses only a partial and most likely unfunctional *nad4L* gene. This is the first time that a functional loss of a *nad* gene not accompanied by the loss of any other core mitochondrial gene has been observed in a plant mitogenome, other than in *Viscum.* The function of the *nad4L* gene product in complex I (mitochondrial NADH dehydrogenase) is also covered by the *nad4* gene product, which in the case of *Krameria lanceolata* is intact.

The other exception is *Phoradendron liga*, another hemiparasitic plant belonging to Viscaceae. Like *Viscum* spp., *P. liga* is a stem parasite and as in *Viscum* a massive gene loss is observed. Using relaxed criteria for gene recognition, Skippington et al. [47] proposed that some of the genes (e.g., *atp4*, *atp8, ccmFc*), which were reported missing in *V. album*, are actually present although very highly divergent. Using the same criteria, presence of additional genes may be proposed in the two other species of *Viscum* previously investigated [26]. Whether these highly divergent sequences do represent homologous and functional genes need further investigation. Irrespective of the precise number of genes present in members of the Viscaceae, massive reduction in gene content remains a fact and a unique feature of the Viscaceae. The closely related hemiparasites *Osyris alba* (root parasite, Santalaceae) and *Loranthus europaeus* (stem parasite, Loranthaceae) both possess all core mitochondrial genes. Thus, at this point there is no evidence of a correlation between level of parasitism and gene contents in the mitogenomes; rather it seems to be highly clade specific. This contrasts to the correlation observed between parasitism and gene loss found in the plastomes of parasitic plants [10, 12, 13].

When it comes to the variable mitochondrial genes there is no clear pattern of gene loss in the parasitic plants. *Phoradendron* has lost most ribosomal protein genes like *Viscum* [26, 27]. For the remaining parasitic plants, six to 14 of the 15 ribosomal protein genes are present. This is not surprising; it has been observed that ribosomal protein genes can be transcribed in the nuclear genome and transferred to the mitochondria [47].

The massive mitochondrial gene loss observed in the Viscaceae [26, 27] remains enigmatic. The possibility that genes are not truly missing from the mitogenome but just unobserved is negligible. For *Phoradendron* and all other taxa investigated in this study, sequencing was done in sufficient depth to capture genes both from the mitogenome and the plastome. However, sequencing depth is not sufficient to reliably detect nuclear genes, thus it remains a possibility that genes missing from the mitogenome could be transferred to the nucleus. Due to the huge size of the *Viscum* nuclear genome (201Gb [48]) it is not a realistic option to increase sequencing depth in order to try to recovered any nuclear copies of the mitochondrial genes. Other methods, e.g., transcriptomics may be more relevant (see below). Thus, the crucial question of how the four *Viscum* species and *P. liga* produce the energy they require in order to sustain life remains, as does the possibility that they have developed a novel mechanism to do so [26].

A eukaryote completely lacking mitochondria was recently characterised by Karnkowska et al. [49]. Thus, an oxymonad species, *Monocercomonoides* sp., has replaced part of the respiratory processes facilitated by mitochondrial genes with others that are suggested to have emerged through lateral gene transfer from bacteria. Also in Archezoa, double membrane-bound, mitochondrion-related organelles were found to harbour genes so modified, that their homology to the standard mitochondrial genes could not be established [50]. *Monocercomonoides* sp. appears to produce ATP through an extended anaerobic glycolysis pathway, coupled with fermentation. However, this scenario seems unrealistic for an organism that actively produces oxygen and has a functional plastome and a photosynthetic apparatus [13]. A few unicellular organisms, e.g., the fungus *Rozella* [51], which have lost the entire complex I genes, use alternative pathways that do not require pumping of protons, but this leads to the production of less ATP in total.

To investigate how *Viscum* and *Phoradendron* survive with their reduced mitogenomes, especially before they establish contact with their hosts (something that may take months in e.g. *Viscum album*), it would be worthwhile to try to capture active mitochondrial RNA molecules using probes that target standard mitochondrial genes as well as the ones identified in the previous studies [26, 27] by transcriptomics. In this manner, it will be possible to verify the presence or absence of the ‘normal’ genes, which may have been missed in the whole genome sequencing approach used here and at the same time also check if the very divergent mitochondrial genes found are active or not. Furthermore, for many of the ‘normal’ genes the 3-dimensional structure of their products is known enabling the targeting of probes to RNA sequences of highly conserved regions.

The evolutionary pathway resulting in the massive mitochondrial gene loss observed in *Viscum* and *Phoradendron* also needs further studies. The Viscaceae is composed entirely of hemiparasitic stem parasites and includes no less than ~ 465 species in six genera, amounting to approximately 20% of all parasitic plants in the Santalales [52], [www.parasiticplants.siu.edu/ListParasites.htm]. Thus, data from additional species and genera of Viscaceae are strongly needed along with data from the closest relatives to Viscaceae, i.e. Amphorogynaceae [2, 53].

### Substitution rates of mitochondrial genes in parasitic plants are not significantly different compared to autotrophic, non-parasitic plants, except for the Viscaceae

In this study, we also wanted to test the hypothesis that parasitic plants have higher substitution rates than autotrophic plants. Early theoretical models have suggested that parasites should evolve much faster than their hosts as a response to the arms race between host and parasite [33]. The highly increased substitution rates of the remaining mitochondrial genes previously shown in *Viscum* [26, 27] are shared and even exceeded by *Phoradendron*, but not by the other representatives of Santalales (Fig. 3). Based on the small data set of 38 taxa it is evident that the holoparasites *Cytinus hypocistis* ssp. *hypocistis* and *C. hypocistis* ssp. *clusii* (Cytinaceae) do indeed have higher substitution rates than their closest autotrophic relative included *Gossypium hirsutum* (Malvaceae) (Fig. 3). The same trend has been observed in their chloroplast genes [54]. However, in other supposedly more distantly related taxa, e.g. *Geranium maderense* (Geraniaceae), we observe notably faster substitution rates than in *Cytinus*. *Pholisma sonorae* (Boraginaceae) evolves slightly faster than *Nicotiana tabacum* L. (Solanaceae)*,* but slower than other representatives of the Lamiids, like *Erythranthe guttata* (Phrymaceae) (Fig. 2). *Lathraea clandestine* and *L. squamaria* (Orobanchaceae) both show elevated substitution rates compared to *E. guttata*, but the non-parasite, *Salvia miltiorrhiza* (Lamiaceae), evolves faster still. The hemiparasitic *Cassytha pubescens* (Lauraceae), *Osyris alba* (Santalaceae), and *Krameria lanceolata* (Krameriaceae) all have similar substitution rates, in the same range as many autotrophic plants. However, within the Santalales, both the holoparasitic *Langsdorffia hypogaea* (Balanophoraceae) and the hemiparasitic *Loranthus europaeus* (Loranthaceae) have higher substitution rates compared to the closely related hemiparasite *Osyris alba* (Santalaceae), although far from the level observed in Viscaceae, where *Phoradendron liga* has the fastest substitution rae of all taxa in the data set.

A previous comparison of parasitic plants with their closest autotrophic relatives showed that the parasites tended to have higher substitution rates across all three genomes [34]. The study compared the substitution rates of four mitochondrial genes (*matR, atp1, nad1* and *cox1)* from 12 parasitic lineages with representatives of the assumed most closely related, available autotrophic lineages, and using pairwise comparisons of the sequences. However, this approach has several pitfalls. Firstly, the phylogenetic position of certain parasitic taxa is ambiguously resolved and the choice of close relatives for comparison thus not necessarily ideal. Further, results can be biased by the exact choice of close relatives from groups where substitution rates vary considerably even among autotrophic members.The Wilcoxon signed-rank test only evaluates pairwise comparisons of two related or matched samples and gives no information as whether the finding is significant for the species, family or group level.

To overcome some of these problems we inferred the phylogenetic placement of all parasitic taxa analysed and calculated substitution rates from the root of the tree, based on the out-group taxa. Furthermore, we performed a one-way analysis of variance on the taxa and groups of taxa as well. The most substantial result is that the Viscaceae, including *Phoradendron liga, Viscum album, V. crassulae, V. minimum* and *V. scurruloideum*, have significantly higher substitution rates of all holoparasitic, hemiparasitic, and autotrophic plants in the 38 taxa data set. There were no statistically significant differences between any of the remaining hemiparasitic plants compared to either the holoparasitic or the autotrophic ones. Several hemiparasitic and holoparasitic plants evolve faster than their closest autotrophic relatives, yet without statically significantly differences. As shown earlier by Bromham et al. [34] *Krameria lanceolata* evolves faster than *Guaiacaum*, however, the difference in mean substitution rates is insignificant (Additional file 6: Table S2). In the Lauraceae, *Cassytha pubescens* has a higher substitution rate than its autotrophic closest relatives, while *Cytinus hypocistis* ssp. *hypocistis* and *C. hypocistis* ssp. *clusii* evolve much faster than their closest relatives (Brassicaceae). The same is observed for *Langsdorffia hypogaea* and *Loranthus europaeus*. On the other hand, *Osyris alba*, the two *Lathraea* species, as well as *Pholisma sonorae* exhibit slower molecular evolution rates than the closely related autotrophs. Thus, there is no evidence that parasitic plants necessarily evolve faster than their closest autotrophic relatives.

An obvious pitfall of the limited taxon sampling in the small dataset of 38 taxa is that we unintentionally may make comparison with either a particular fast or slowly evolving relative, hence biasing the results. To overcome this problem we expanded our data set to a total of 418 taxa but only four genes making use of the data matrix by Qiu et al. [41] (including a few duplicates as a means of verification of similar sequences used in the analysis). Running a group- or clade-specific (Viscaceae, holoparasitic, hemiparasitic, autotrophic plants) one-way analysis of variance of all the taxa of the large data set we again found the Viscaceae to have a significantly faster substitution rate than any other group of holoparasitic, hemiparasitic, and autotrophic taxa. However, running the same analysis this time comparing each parasitic taxon to the rest and not grouping them into categories we made some unexpected observations. While *Phoradendron* and three of the *Viscum* species still had significantly higher substitution rates, *V. album* did not (Additional file 6: Table S2). The explanation for this seems to be the difference in gene content of the five Viscaceae species. All species possess the fast evolving *atp1* gene, all lack *nad5*, *V. album* and *V. scurruloideum* possess *rps3*, but only *V. album* possess *matR*, which in this species has a low substitution rate. Thus, the presence of *matR* lowers the overall substitution rate to a similar level as observed in several autotrophic taxa like *Acorus, Hydrolea* and *Geranium.* Similarly, the remaining holoparasitic and hemiparasitic taxa of the analysis exhibit mean evolution rates that fall within the same range as the autotrophic taxa. Thus, there is no statistically significant difference between the remaining taxa, equal to what we observed within the group-specific one-way analysis of variance. Even parasitic plants that have eliminated photosynthesis and lost most or all of their plastid genes still maintain their mitochondrial genes with only minor alterations.

Considering the suggestion that parasites should evolve much faster than their hosts as a response to an arms race between host and parasite [33], a comparison of substitution rates between hosts and parasites would appear relevant. However, many parasitic plants have a worldwide distribution and parasitize on a variety of hosts belonging to different species, genera, or even families (e.g., *Viscum album* is known to parasite 367 hosts – including species, subspecies and varieties, 97 genera and 46 families [55]). Moreover, in certain cases – especially among root parasites – it can be exceedingly difficult to locate the host of the parasite with certainty. Thus, the remark based on what is observed from bacteria in regards to arms race between the parasite and the host cannot easily be tested in plants. However, the global analysis using as many taxa as possible as conducted here does not support the postulate with regards to mitochondrial gene evolution.

### Some Phylogenetic Observations

Holoparasites very often have a strongly reduced and modified morphology, which makes them very difficult to place in a phylogenetic context [56]. Additionally they usually have a strongly reduced and divergent plastome that have made it equally difficult to place them in a molecular phylogenetic context as most major phylogenies are based on plastome genes [57, 58] or whole plastomes [59]. The current phylogenetic analyes based only on mitochondrial genes, may thus provide further evidence on the relationships of these parasitic taxa.

Here, we find *Langsdorffia* embedded in the Santalales (Fig. 2; Additional file 4: Figure S1). This is consistent with the recent phylogenetic analysis of the Santalales, where three plastid genes (*matK, rbcL* and *accD*), one mitochondrial gene (*matR*), and three nuclear loci (SSU rDNA, LSU rDNA and *rpb2*) were used to demonstrate the placement of the Balanophoraceae within the order [2]. Su et al. [2] further observed that the Balanophoraceae is non-monophyletic consisting of two clades with different relationships. The genus *Langsdorffia* was not included in their analysis, but due to the much less dense taxon sampling of Santalales in our analyses and some differences in tree topology, we cannot place *Langsdorffia* in either of the clades. However, we do provide independent evidence for the inclusion of Balanophoraceae in Santalales.

In the present study, we have performed two widely different phylogenetic analyses: one with a shallow taxon sampling, but massive data sampling (38 taxa, 40,091 characters), and one with very dense taxon sampling, but modest data sampling (418 taxa, 9076 characters). Compared to the general conception of angiosperms phylogeny as represented by APG IV [60], the latter data set results in a considerably more accurate phylogenetic tree. This observation is in full accordance with the general importance of density in taxon sampling as discussed by e.g. Heath et al. [61], and Nabhan and Sarkar [62].

However, while some of the parasitic taxa, which seem misplaced in the analysis using few taxa (i.e. *Cassytha*, *Krameria*), are recovered in expected positions when using many taxa, some remain in unexpected positions. *Cynomorium*, which most likely belongs to the Saxifragales, is placed as sister to a clade including all superasterids, however this position lacks bootstrap support. Further, we recover the Viscaceae clade, which would also be expected to be included in the Santalales, within a rather strongly supported Ericales clade (Additional file 4: Figure S1). This position is surprising since the phylogenetic inclusion of Viscaceae in Santalales has been demonstrated repeatedly and never questioned (e.g. [2, 53]). However, Skippington et al. [47] recently suggested that the *matR* gene present only in *V. album*, but not in any other Viscaceae, had been required through horizontal gene transfer from a member of the Ericales. Although the evidence is circumstantial this could potentially explain the position seen even here in our four-gene analysis, since the only other sequence data which are included for the Viscaceae taxa are the highly divergent *atp1* sequences.

## Conclusions

In this project, we sequenced and analysed 11 parasitic plants spread throughout the angiosperm phylogeny. We show that the Viscaceae are truly unique among parasitic plants both in regards to their mitochondrial gene content and their mitochondrial gene evolution, having the most reduced and fastest evolving mitogenomes to date. We also show that the mitogenomes of parasitic plants in general do not evolve faster than that of their autotrophic relatives. There are several autotrophic taxa that have significantly higher evolutionary rates in their mitochondrial genes than the parasites. Comparison of the substitution rates of the mitogenomes of holoparasitic, hemiparasitic, and autotrophic plants shows no significant difference. This goes hand in hand with the fundamental role of the mitogenome in respiration, a feature of high importance, commonly shared across all plant taxa. Lastly, we pinpointed the phylogenetic position of *Langsdorffia hypogaea* (Balanophoraceae) in the Santalales.

## Additional files

Additional file 1: **Table S1.** Tissue type, protocol, cleaning procedures and mass used for DNA extraction of the 11 samples analysed in this study. (DOCX 57 kb)

Additional file 2: **Table S4.** GeneBank Accession Numbers of the non-parasitic taxa used in the small dataset of 38 taxa. (XLS 104 kb)

Additional file 3: Code C1: R code used for the analyses of variance in the small and large data set. (DOCX 12 kb)

Additional file 4: **Figure S1.** Phylogenetic tree of the 418 taxa included in the study. Branch length shows substitution rates, while bootstraps support values are shown on the base of each branch. (PDF 682 kb)

Additional file 5: **Table S3.** ANOVA of the substitution rates in the data set of the 38 taxa for the: a) *atp1* gene, b) *matR* gene, c) *rsp3* gene and d) all_mt_genes. (PDF 114 kb)

Additional file 6: **Table S2.** ANOVA of the substitution rates in the data set of 418 taxa using 4 mitochondrial genes (analysis 4). (XLSX 41 kb)

## Abbreviations

ANOVA: Analysis of variance
APG: Angiosperm phylogeny group
BS: Bootstraps support
LSU: Large subunit
mt: Mitochondrial
SSU: Small subunit
