# Supplementary material for: Mitochondrial genome evolution in parasitic plants
Source: BMC Evol Biol. 2019 Apr 8;19:87. doi: 10.1186/s12862-019-1401-8 (PMC6454704; doi:10.1186/s12862-019-1401-8)
Supplement: Supplementary file 1 — Table S1. Tissue type, protocol, cleaning procedures and mass used for DNA extraction of the 11 samples analysed in this study. (DOCX 57 kb) [file 12862_2019_1401_MOESM1_ESM.docx]

|  | | |  |  |  |  |  |  |
| --- | --- | --- | --- | --- | --- | --- | --- | --- |
| **#** | **Project ID** | **Name** | | | | **Tissue type** | **Cleaning/crushing** | **Mass (g)** |
| 1 | **C3120** | *Sapria himalayana* | | | | Flowers | no cleaning | 0.013 |
| 2 | **C3122** | *Pholisma sonorae* | | | | Stem and flowers | no cleaning | 0.027 |
| 3 | **C3123** | *Cassytha pubescens* | | | | Stem and flowers | no cleaning | 0.018 |
| 4 | **C3124** | *Krameria lanceolata* | | | | Stem and leaves | no cleaning | 0.016 |
| 5 | **C3126** | *Langsdorffia hypogaeae* | | | | Leaf | rinsing with H2O | 0.006 |
| 6 | **C1935** | *Lathraea squamaria* | | | | Stem and flowers | no cleaning | 0.035 |
| 7 | **C3089** | *Lathraea clandestina* | | | | Flowers | no cleaning | 0.05 |
| 8 | **C3127** | *Loranthus europaeus* | | | | Stem | no cleaning | 0.061 |
| 9 | **C3151** | *Cynomorium coccineum* | | | | Stem | no cleaning | 0.079 |
| 10 | **C3152** | *Cytinus hypocistis* ssp*. clusii* | | | | Stem and flowers | no cleaning | 0.029 |
| 11 | **C3158** | *Cytinus hypocistis* ssp*. hypocystis* | | | | Stem and flowers | no cleaning | 0.033 |

**Supplementary material Table S1**
