# Supplementary material for: Mitochondrial genome evolution in parasitic plants
Source: BMC Evol Biol. 2019 Apr 8;19:87. doi: 10.1186/s12862-019-1401-8 (PMC6454704; doi:10.1186/s12862-019-1401-8)
Supplement: Supplementary file 3 — Code C1: R code used for the analyses of variance in the small and large data set. (DOCX 12 kb) [file 12862_2019_1401_MOESM3_ESM.docx]

Supplementary materials Code C1

library(MASS)

library(ape)

library(faraway)

library(agricolae)

data<-read.csv(file.choose(), header = TRUE)

data

m1<-lm(Substitution_rate~Taxon, data=data)

shapiro.test(data$Substitution_rate)

boxplot(Substitution_rate~Taxon, data=data)

anova1<-aov(m1)

summary(anova1)

LSD<-LSD.test(anova1,"Taxon",p.adj="bon",console=TRUE)

summary(LSD)

par(mar=c(5,1,1.5,0.5),cex=0.5)

HSD1<-HSD.test(anova1,"Taxon",p.adj="bon",console=TRUE)

bar.group(HSD1$groups,ylim=c(0,2),density=30,border="black", lwd= 1, las=2, xlab="Taxa", ylab="Substitutions per site", main="Substitution rates of mitochondrial genes in angiosperms")
