# Supplementary figures and images for: Mitochondrial genome evolution in parasitic plants
Source: BMC Evol Biol. 2019 Apr 8;19:87. doi: 10.1186/s12862-019-1401-8 (PMC6454704; doi:10.1186/s12862-019-1401-8)

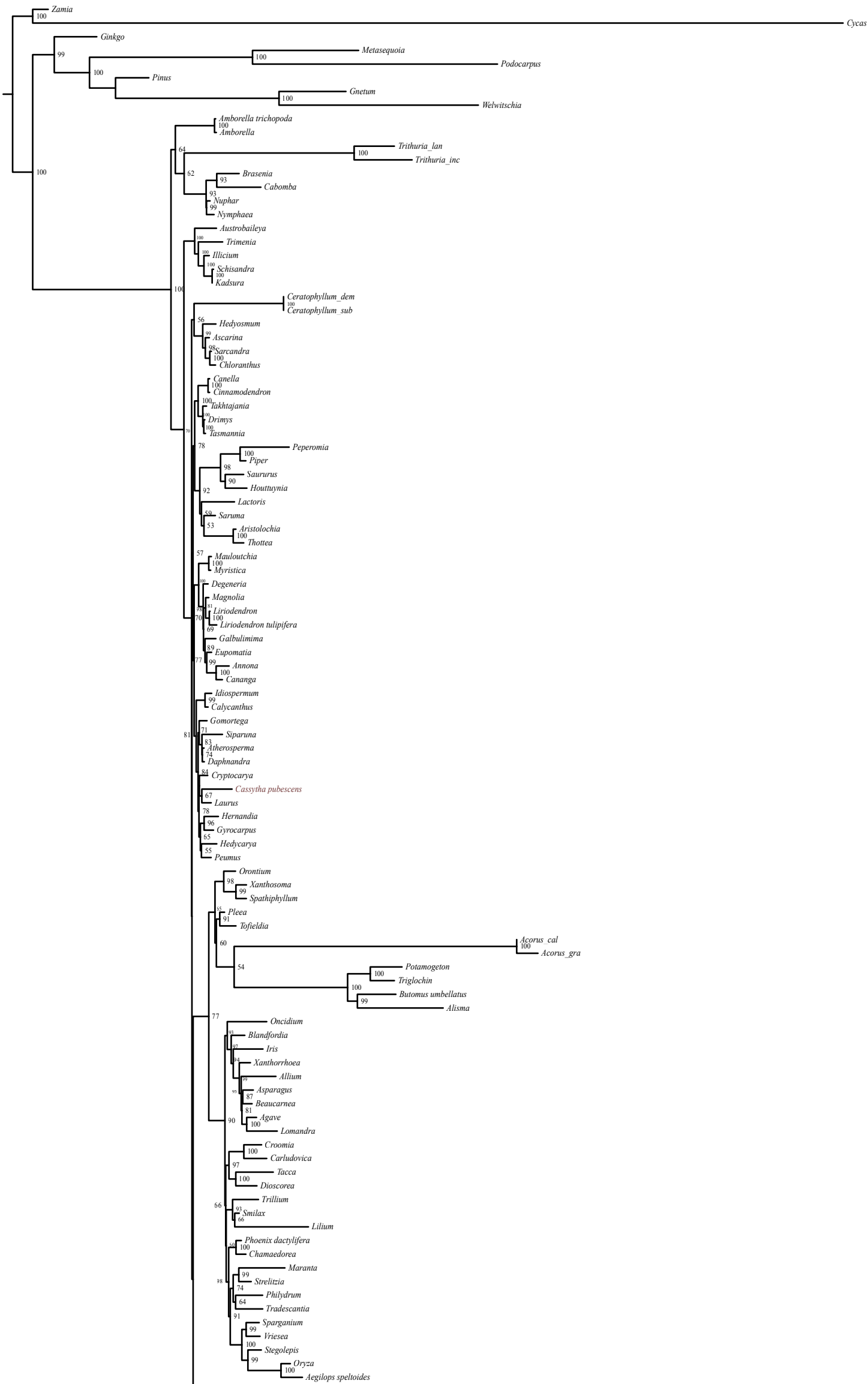

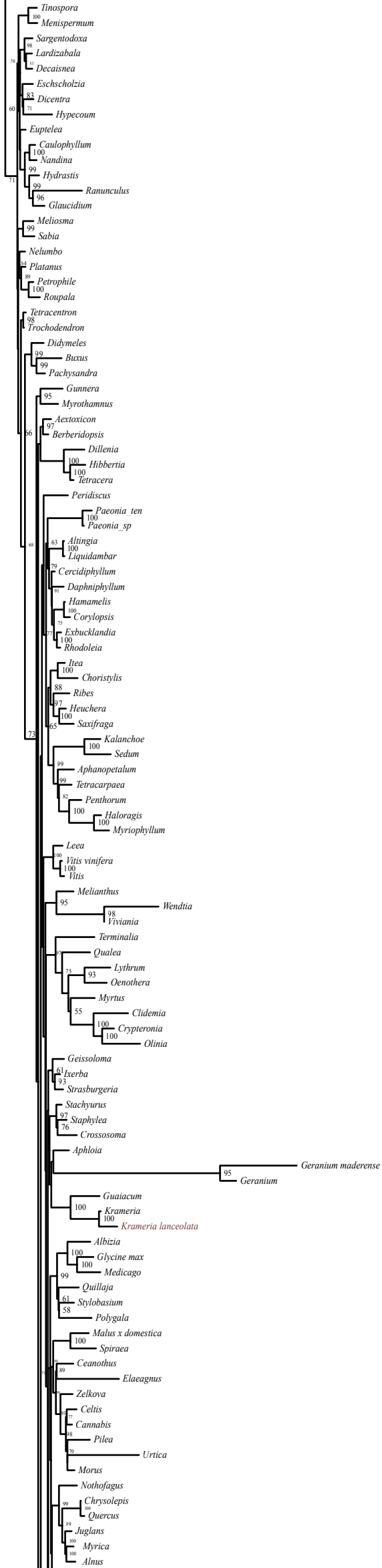

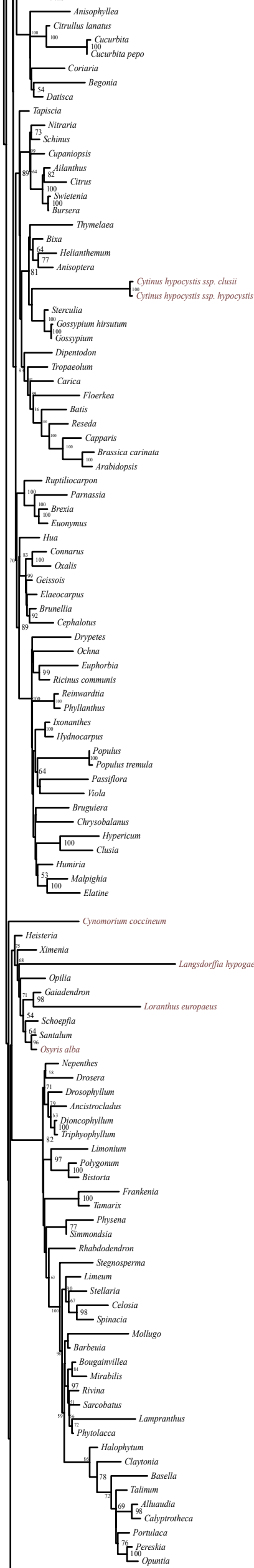

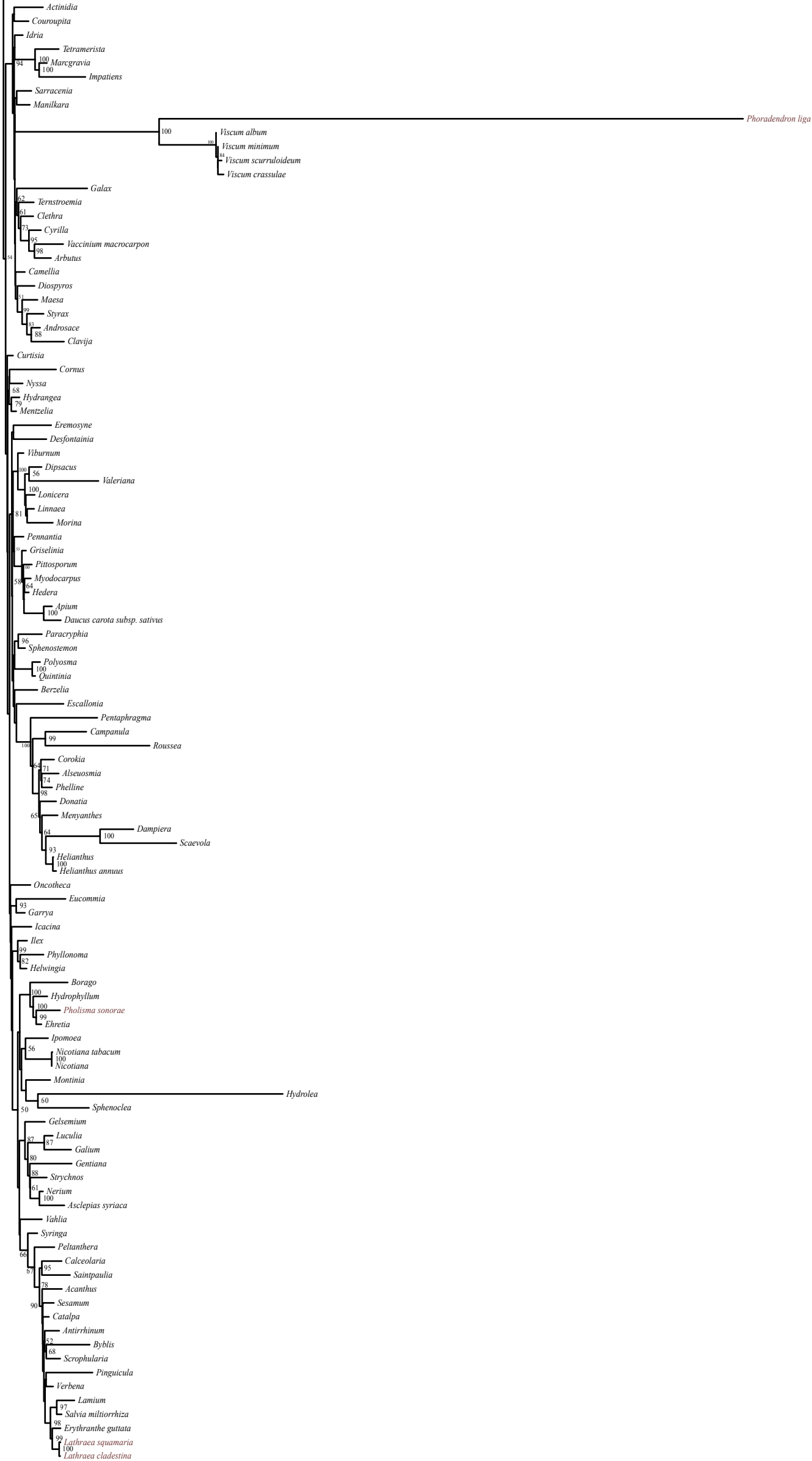

Supplement: Supplementary file 4 — Figure S1. Phylogenetic tree of the 418 taxa included in the study. Branch length shows substitution rates, while bootstraps support values are shown on the base of each branch. (PDF 682 kb) [file 12862_2019_1401_MOESM4_ESM.pdf]
