# Supplementary material for: Mitochondrial genome evolution in parasitic plants
Source: BMC Evol Biol. 2019 Apr 8;19:87. doi: 10.1186/s12862-019-1401-8 (PMC6454704; doi:10.1186/s12862-019-1401-8)
Supplement: Supplementary file 5 — Table S3. ANOVA of the substitution rates in the data set of the 38 taxa for the: a) atp1 gene, b) matR gene, c) rsp3 gene and d) all_mt_genes. (PDF 114 kb) [file 12862_2019_1401_MOESM5_ESM.pdf]

RAXML *atp1*

Taxon	Substitutions per site
Amborella trich	0.45
Liriodendron tu	0.462761
Butomus umbel	0.713119
Aegilops speltoi	0.518501
Phoenix dactyli	0.476673
Vitis vinifera	0.517358
Glycine max	0.55606
Malus x domest	0.533928
Citrullus lanatu	0.516275
Cucurbita pepo	0.538396
Ricinus commu	0.516152
Populus tremul	0.648369
Geranium mad	0.613709
Brassica carina	0.559698
Gossypium hirs	0.530747
Vaccinium mac	0.573184
Daucus carota	0.540606
Helianthus ann	0.532641
Nicotiana tabac	0.534591
Asclepias syria	0.569102
Salvia miltiorrh	0.598394
Erythrante gutt	0.596085
Cassytha pubes	0.510899
Krameria lance	0.541703
Osyris alba	0.528879
Loranthus euro	0.553731
Cytinus hypocis	0.582777
Cytinus hypocis	0.582777
Lathraea squan	0.599447
Lathraea clades	0.59445
Langsdorffia hy	0.578592
Cynomorium co	0.546648
Pholisma sonor	0.538226
Phoradendron l	1.681055
Viscum album	1.377452
Viscum crassul	1.382571
Viscum scurrul	1.380518
Viscum minimu	1.381541

1-way ANOVA

4 independent groups: non-parasitics, hemiparasitic, holoparasitic, Viscacae

Total number of substitutions (RAXML with Viscacae)

Data summary

	Samples 1	2	3	4	**Total**
N	22	4	7	5	38
Σx	12.096349	2.128129	4.03	7.203137	25.457615
Mean	0.549834	0.532032	0.575714	1.440627	0.669937
Σx2	6.725545	1.132997	2.32304	10.449308	20.630889
Variance	0.00355	0.000255	0.000485	0.018068	0.096646
Std. Dev.	0.059586	0.015955	0.022026	0.134417	0.310879
Std. Error	0.012704	0.007978	0.008325	0.060113	0.050431

Standard weighted-means analysis

ANOVA summary Independent samples k=4

Source	SS	dF	MS	F	P
Treatment [Between groups]	3.425	3	1.142	257.94	<.0001
Error	0.151	34	0.004427		
Ss/BI					
Total	3.575885	33			

Tukey HSD Test

HSD[.05]=0.1; HSD[.01]=0.13

Non-parasitic vs Hemiparasitic	nonsignificant
Non-parasitic vs Holoparasitic	nonsignificant
Non-parasitic vs Viscaceae	p < .01
Hemiparasitic vs Holoparasitic	nonsignificant
Hemiparasitic vs Viscaceae	p < .01
Holoparasitic vs Viscaceae	p < .01

RAXML *matR*

Taxon	Substitutions per site
Amborella trich	0.45
Liriodendron tu	0.463694
Butomus umbel	0.549789
Aegilops speltoi	0.511798
Phoenix dactyli	0.47909
Vitis vinifera	0.471638
Glycine max	0.492041
Malus x domest	0.490286
Citrullus lanatu	0.496434
Cucurbita pepo	0.525366
Ricinus commu	0.490335
Populus tremul	0.503155
Geranium mad	0.523952
Brassica carina	0.506726
Gossypium hirs	0.485895
Vaccinium mac	0.499895
Daucus carota	0.489074
Helianthus ann	0.492351
Nicotiana tabac	0.496596
Asclepias syria	0.502191
Salvia miltiorrh	0.492975
Erythrante gutt	0.494817
Cassytha pubes	0.468484
Krameria lance	0.49572
Osyris alba	0.47698
Loranthus euro	0.520245
Cytinus hypocis	0.534492
Cytinus hypocis	0.534492
Lathraea squan	0.490664
Lathraea clades	0.490664
Langsdorffia hy	0.600034
Cynomorium co	0.500293
Pholisma sonor	0.495376
Phoradendron liga	
Viscum album	0.491559
Viscum crassulae	
Viscum scurruloideum	
Viscum minimum	

1-way ANOVA

4 independent groups: non-parasitics, hemiparasitic, holoparasitic, Viscacae

Total number of substitutions (RAXML with Viscacae)

Data summary

	Samples 1	2	3	4	**Total**
N	22	4	7	1	34
Σx	10.908098	1.941477	3.665967	0.491559	17.007101
Mean	0.495823	0.485369	0.52371	0.491559	0.500209
Σx2	5.417782	0.943019	1.928959	0.24163	8.531389
Variance	0.000443	0.000228	0.001509	NA	0.000736
Std. Dev.	0.021044	0.15115	0.038852	NA	0.027129
Std. Error	0.004487	0.007557	0.014685	NA	0.004653

Standard weighted-means analysis

ANOVA summary Independent samples k=4

Source	SS	dF	MS	F	P
Treatment [Between groups]	0.005	3	0.002	2.75	0.06003
Error	0.019	30	0.000635		
Ss/BI					
Total	0.024	33			

Tukey HSD Test

This test will be performed only if K>2 and the analysis of variance yields a significant F-ratio.

RAXML *rps3*

Taxon	Substitutions per site
Amborella trich	0.45
Liriodendron tu	0.465145
Butomus umbel	0.596686
Aegilops speltoi	0.526784
Phoenix dactyli	0.485985
Vitis vinifera	0.477226
Glycine max	0.49928
Malus x domest	0.49911
Citrullus lanatu	0.492865
Cucurbita pepo	0.514111
Ricinus commu	0.487673
Populus tremul	0.537351
Geranium mad	0.879598
Brassica carina	0.537274
Gossypium hirs	0.509638
Vaccinium macrocarpon	
Daucus carota	0.527862
Helianthus annuus	
Nicotiana tabac	0.490121
Asclepias syria	0.501946
Salvia miltiorrh	0.510934
Erythrante guttata	
Cassytha pubes	0.478239
Krameria lance	0.500003
Osyris alba	0.482563
Loranthus euro	0.647872
Cytinus hypocis	0.579527
Cytinus hypocis	0.576756
Lathraea squan	0.507129
Lathraea clades	0.508351
Langsdorffia hy	0.567996
Cynomorium co	0.531116
Pholisma sonor	0.519725
Phoradendron liga	
Viscum album	
Viscum crassulae	
Viscum scurruloideum	
Viscum minimum	

1st 1-way ANOVA

3 independent groups: non-parasitics, hemiparasitic, holoparasitic

Substitution rates (RAXML without Viscacae)

Data summary

	Samples 1	2	3	**Total**
N	19	4	7	30
Σx	8.976709	1.991921	3.907356	14.875986
Mean	0.472458	0.49798	0.558194	0.495866
Σx2	4.889454	0.993667	2.196571	8.079692
Variance	0.036018	0.000576	0.002585	0.024248
Std. Dev.	0.189785	0.02401	0.050842	0.155718
Std. Error	0.04354	0.012005	0.019217	0.02843

Standard weighted-means analysis

ANOVA summary Independent samples k=3

Source	SS	dF	MS	F	P
Treatment [Between groups]	0.038	2	0.019	0.76	0.477411
Error	0.665518	27	0.024651		
Ss/BI					
Total	0.703	29			

Tukey HSD Test

This test will be performed only if K>2 and the analysis of variance yields a significant F-ratio.

RAXML all_mt_genes

Taxon	Substitution rate
Amborella trich	0.45
Liriodendron tu	0.460027
Butomus umbel	0.546915
Aegilops speltoi	0.528624
Phoenix dactyli	0.491331
Vitis vinifera	0.48716
Glycine max	0.51054
Malus x domest	0.502548
Citrullus lanatu	0.500209
Cucurbita pepo	0.520435
Ricinus commu	0.501963
Populus tremul	0.52545
Geranium mad	0.592562
Brassica carina	0.525093
Gossypium hirs	0.511083
Vaccinium mac	0.517457
Daucus carota	0.531828
Helianthus ann	0.52933
Nicotiana tabac	0.507866
Asclepias syria	0.522195
Salvia miltiorrh	0.548203
Erythrante gutt	0.518825
Cassytha pubes	0.484363
Krameria lance	0.513483
Osyris alba	0.512532
Loranthus euro	0.55266
Cytinus hypocis	0.569217
Cytinus hypocis	0.561156
Lathraea squan	0.521048
Lathraea clades	0.522013
Langsdorffia hy	0.569032
Cynomorium co	0.5209
Pholisma sonor	0.511378
Phoradendron l	1.621477
Viscum album	1.198901
Viscum crassul	1.254727
Viscum scurrul	1.209843
Viscum minimu	1.203001

1-way ANOVA

4 independent groups: non-parasitics, hemiparasitic, holoparasitic, Viscacae

Substitution rates (RAXML with Viscacae)

Data summary

	Samples 1	2	3	4	**Total**
N	22	4	7	5	38
Σx	11.329	2.031278	3.8065	6.487949	23.655375
Mean	0.5149	0.50782	0.5437	1.29759	0.62251
Σx2	5.85	1.0322	2.0736	8.551823	17.510671
Variance	0.000873	0.000259	0.000618	0.033282	0.075269
Std. Dev.	0.029549	0.016079	0.0248	0.182432	0.274353
Std. Error	0.0063	0.007862	0.009393	0.081586	0.044506

Standard weighted-means analysis

ANOVA summary Independent samples k=4

Source	SS	dF	MS	F	P
Treatment [Between groups]	2.629	3	0.876	191.07	<.0001
Error	0.156	34	0.004587		
Ss/BI					
Total	2.785	37			

Tukey HSD Test

HSD[.05]=0.1; HSD[.01]=0.13

Non-parasitic vs Hemiparasitic	nonsignificant
Non-parasitic vs Holoparasitic	nonsignificant
Non-parasitic vs Viscaceae	p < .01
Hemiparasitic vs Holoparasitic	nonsignificant
Hemiparasitic vs Viscaceae	p < .01
Holoparasitic vs Viscaceae	p < .01
